# Dichotomy of Tenure and Biomedical Engineering Research with a Purpose in an Academic Setting

**DOI:** 10.3389/fped.2015.00113

**Published:** 2015-12-23

**Authors:** Esra Roan

**Affiliations:** ^1^University of Memphis, Memphis, TN, USA

**Keywords:** tenure, academia, PhD, career, STEM

I am one of the many biomedical engineering researchers whose work is built on strong interactions with physicians and basic scientists who find value in the way we approach healthcare problems from alternate perspectives. For example, I collaborated with multiple lung critical care physicians in the past 5 years, one of whom was a pediatric critical care physician at a children’s hospital in Memphis, TN, USA. We met regularly in lung physiology research meetings organized by our mentor to discuss problems relating to pediatric lung health, and we carried out research activities to understand the role of mechanobiology of potassium channels in the context of mechanical ventilation (1, 2). In addition to these types of research collaborations, biomedical engineers in academia contribute to the healthcare of pediatric patients also via design initiatives. Just yesterday, I listened to a set of senior design projects that displayed prototypes of at-home mechanical ventilator systems for young children aimed to increase their mobility. These projects were based on collaborations established between the therapists, clinicians, and professors at multiple institutions (Le Bonheur Children’s Hospital, University of Memphis, University of Tennessee Health Science Center). As such, most would agree with me in saying that these collaborations are important and necessary to develop and optimize healthcare solutions.

We, on the biomedical research side, are aware of and sensitive to the issues clinicians are facing with regards to work–life balance and dropout. Here, I want to present that there are similar concerns in academic biomedical research as well. They stem from similar stressors relating in great part to the early career hiring practices. Particularly, academic research in biomedical engineering is highly demanding and competitive in large part due to a pressure cooker entry period: tenure. Any conversation regarding work–life balance in academia must include the long lasting scars these junior years leave in our lives and careers, whether we succeed or fail. In the years of the “probationary” period toward tenure, an assistant professor is asked to teach, research, publish, and raise funds. Depending on the university, these may carry different weights, but in this newer academic climate, where the support for higher education is declining, fundraising is a top priority. In order to secure funding, we write grant applications with research ideas that sound non-risky to reviewers that are curbed versions of our original ideas (3). In order to become fundable, we publish as many papers as quickly as we can. All of these add up to countless hours of work countered by numerous critical reviews, which we learn slowly to accept, read, and respond. Although one can conduct biomedical research outside of academia, many believe in the strong inter-disciplinary research between medical schools and engineering colleges that it can provide the answers to some of our greatest healthcare problems. Thus, many of us chose an academic career over the more lucrative industrial careers for the intellectual freedoms and the possibility of finding meaningful careers through teaching and research.

A stable career in biomedical research is rewarded after the successful completion of a race, a *marathon*, which includes many forms of training years followed by the time-limited probationary tenure period. We do not perceive it as a race until a few years into the tenure process, we realize that it is indeed a race against time. Once the elation of securing a highly competitive tenure-track position dissipates, the perception of the race solidifies. Submission of grants, number of publications, funds generated, and students graduated become markers of success. In rare moments of calm between submissions, graduations, and evaluations, small amounts of doubt about the purpose underlying day-to-day activities creeps in, but it is erased quickly as the momentum generated at the beginning of the race moves us forward *mindlessly*.

As we get our tenure clock started, what is not explicit in our minds is that the tenure period lines up with the time that we form families or start setting roots within our communities. The former is key to academic careers, especially for female faculty members. It is reported that family formation is a negative factor for women in achieving tenure, whereas it has a net positive impact on men’s tenure prospects (4). In fact, an assistant professor who is female and has children works 100-plus hours a week in all parts of her life while dedicating 52.5 h per week to her academic work, whereas a male assistant professor works overall 88 h per week overall, of which 56.3 h go toward professional duties (5). As the definition of the modern family evolves, it is certain that these numbers will change. I can certainly speak of my experience, which has been on target with the overall number of hours worked in a week, but I was able to dedicate more time to professional activities due to my husband’s major contributions to parenting and household management.

While we are on our tenure race, as we aim to balance all aspects of life on our shoulders, we begin to sense the reality of contemporary academic job description, which primarily involves external fundraising. This mindset is especially prevalent in engineering colleges due to the fact that many consider engineering to be lucrative in raising funds, specifically, biomedical engineering, as there is a high level of investment by the private sector in biotechnology development and innovation. Inadvertently, it is even difficult to admit that these lead many early career researchers to work toward meeting *target metrics* to attain tenure, and possibly lose touch with the purpose of the work that attracted them to the field.

Is it possible that working mindlessly toward tenure in this academic climate is the major reason fueling work–life balance inquiries in biomedical research at mid-career, even after tenure? Although philosophical in nature, this question has real life implications such as the low *retention* rates of female faculty or female workforce in science, technology, engineering, and mathematics (STEM) all together. A survey of global science, engineering, and technology companies in a Harvard Business Review Report revealed that more female engineers, scientists, and technologists quit their jobs mid-career, about 10 years in, compared to male counterparts (6). Another study showed that approximately 50% women in STEM left their careers by the 12th year, whereas only 20% of the women in other professional occupations exited their career by the end of a 30 year span (7). Meanwhile, a similar trend was shown for academia indicating the presence of fewer full professor women than assistant professor ones in the STEM fields (4). Although a multitude of reasons are provided elsewhere, one finding stands out that women faculty are less satisfied than male faculty (8). The source of this dissatisfaction can be attributed to factors such as the environment, lack of role models, etc. Interestingly, the dissatisfaction and dropout may also be associated with one innate difference in how men and women approach their work and career: women place higher value on work that contributes to the society having a *purpose* (9).

This line of thinking suggests that the problem is not a work–life balance issue because there is none when we are dedicated to our work! The underlying issue is whether we perceive the work to which we are dedicating a substantial part of our life is worth doing so. If it is meaningful, then work and life activities *integrate* naturally, which leads to greater overall satisfaction and higher productivity as reflected in Dr. Langer’s work (10). Clinicians and physicians have direct interactions with patients whose lives they impact and save. In the academic biomedical research, we may never see the fruits of our work making a difference in someone’s life. As the demands of tenure mount and the morale in academic workforce drops with each new manufactured metric of institutional success, we find that our day-to-day work contributes less to the society; we begin to raise concerns over work–life balance and wonder if all of what we do is worth the personal cost.

While we await far-reaching solutions, such as increasing science funding and institutional modernization of the tenure and promotion process, we can do some simple things to help with these issues encountered by some. We can begin by examining the solutions suggested for the STEM women in the industry. Among them are job sharing, flexible careers, extended maternity leaves, sideways moves in careers. Implementation of some of these solutions may require changing of the tenure process or eliminating it all together, neither of which will occur in the near future. One answer may be to provide alternative, non-tenure track, sustainable academic research careers where one can expect long-term employment rather than the usual 2-year post-doctoral or research assignments that are highly dependent on external funding obtained by the principal investigator. This unstable situation of employment and income leaves many young researchers, some of whom are supporting families, frustrated and leads them to alternate careers.

Another important aspect in this conversation is the education of pre-doctoral students about an academic career in biomedical engineering research. For example, I was not aware that beginning of a career in biomedical research is similar to starting a small company, where one has to devise and market ideas, become proficient in finance, learn to manage people, etc. Moreover, up until 1 year into my first academic position as a research assistant professor (non-tenure track), I did not know much about the fact that academic research careers are extremely *competitive*, more so than most industrial careers in my field! As such, I suggest that until the funding levels increase and tenure is modernized, we inform and train younger engineering scientists to face these challenges. My interactions with PhD students in the biomedical research field tell me that many students do not understand the job description of a contemporary faculty member in the field. As a junior faculty who just went through the tenure process, I think this information *must* be presented to the students alongside training that involves budgeting, time management skills, etc. Above all, I wholeheartedly feel that the information has to be delivered in a mindful way through mentoring activities and conversations, where realistic undertones prevail over pessimistic ones.

For some of us, work–life balance questions resonate because we are unhappy with the current system. In the meantime, we are thankful to have the opportunity of tenure or a job in biomedical engineering research, which could be considered a form of art where we utilize math and sciences as our media to generate novel solutions that improve healthcare of patients. The essence of this work is intense, creative, and purposeful. However, the system is now skewed such that it is built on funding dollars and numbers met in institutional metrics. I am, for one, an engineer driven by numbers, and I believe that we as faculty should be measured and accountable for the ways we serve the community. The question is: how? When your aim is to understand how the alveolar epithelial cells in the lung are injured with mechanical ventilation to save patients with ARDS, what metrics should you use to measure the success of this research? Number of papers published? Amount of external research dollars? So long as these remain the metrics used to measure our work leading to tenure and promotion, they will also lead to discontent in academia and to dropout at the end. There is also a strong possibility that these metrics are particularly damaging to careers of women who are shown to value purposeful work that benefits the society more than male counterparts. In conclusion, I think that adjusting the new institutional metrics to reflect quality and purposeful work would significantly lower the work–life balance issues associated with biomedical research in academia and lead to less dropout rates in STEM academic fields in general. Whether this is achievable remains to be seen.

## Conflict of Interest Statement

The author declares that the research was conducted in the absence of any commercial or financial relationships that could be construed as a potential conflict of interest.
